# Revitalising Evidence-based Policy for the Sendai Framework for Disaster Risk Reduction 2015-2030: Lessons from Existing International Science Partnerships

**DOI:** 10.1371/currents.dis.aaab45b2b4106307ae2168a485e03b8a

**Published:** 2015-04-23

**Authors:** Elizabeth Carabine

**Affiliations:** Climate and Environment Programme, Overseas Development Institute, London, United Kingdom

## Abstract

The convergence of agreements on disaster risk reduction (DRR), development finance, sustainable development and climate change in 2015 presents a unique opportunity for coherence across these inter-related policy areas. At the same time, demand is growing for a more prominent and effective role for science and technology in providing evidence for policy, with the international community recognising that successful disaster risk reduction (DRR) depends on it. Reflecting this ambition, science is included as a core aspect of the Sendai Framework for Disaster Risk Reduction 2015-2030, although the ways in which this will be implemented in practice is still unclear. This paper aims to inform the implementation of international science coordination for DRR by examining a number of existing international science partnerships used across other relevant areas of policy to understand best practice, options for coordination and lessons identified. In the field of DRR, the science-policy interface needs to be strengthened in line with the best practice described in this review. An enhanced UNISDR Scientific and Technical Advisory Group will be given the mandate for to enhance the evidence base for DRR and mobilise science and technical work in coordination with a broad range of stakeholders. The structure and function of an enhanced STAG must be as open, as inclusive and as participatory as possible in order to build trust in new and existing institutions at local, national, regional and global levels. The challenge for the international community is to facilitate evidence-based policy making by formally recognising the links between DRR, development finance, sustainable development and climate change in the upcoming post-2015 agreements.

## Introduction

Climate change, sustainable development, development finance and disaster risk reduction policies are entering a new phase. Throughout 2015, governments will attend a series of meetings to agree new international frameworks, the first of which was the Third World Conference on Disaster Risk Reduction in Sendai, Japan in March. Next on the agenda are: the Third International Conference on Financing for Development in Addis Ababa, Ethiopia in July; the Sustainable Development Goals will be agreed in September; and, the United Nations Convention for Climate Change Conference of Parties in Paris, France in December. The convergence of these agreements presents a unique opportunity for coherence across these inter-related policy challenges in the post-2015 era.

At the same time, the role of science and technology in providing evidence for policy is gaining prominence, with demand growing for multidisciplinary enquiry to address the complex and inter-related problems of climate change, disasters and sustainable development^1^^,^ 2 ^,^ 3. Innovations in methods, tools and analyses have made significant leaps in finding solutions, and more data is becoming widely accessible^4^. The communication of this scientific evidence to policymakers increasingly is becoming a key challenge^5^. Also there is a recognised need for international science partnerships to provide more than assessments of scientific information, particularly where different kinds of knowledge can contribute solutions, for more explicit links to decision making^6^. The need for capacity-building for different kinds of actors is paramount to ensure policy support can be provided^6^^,^ 7. With the changing nature of the science-policy interface, science advisory services and initiatives must evolve to meet these challenges.

The critical role and value of scientific information and technology for successful disaster risk reduction (DRR) and resilience has been recognised by the international community. As such, science is included as a core aspect of the Sendai Framework for DRR 2015-2030^8^, although the ways in which this will be implemented in practice is still unclear. It has been recommended that DRR requires scientific and technical capacities with inputs from natural, environmental, social, economic, health and engineering disciplines^9^ and particularly needs participation of practitioners as well as academics^2^. Only 11 countries have a government chief scientific advisor with a mandate to interface directly between science and technology and policy makers^2^^,^ 10, but worldwide there are 107 national scientific academies/institutions mandated with DRR or disaster risk management^11^. There is an urgent need at the global level for enhanced partnership and coordination for evidence-based approaches within the Sendai Framework.

This paper aims to inform the implementation of the science coordination aspects of the Sendai Framework by examining a number of existing international science partnerships used across other relevant areas of policy to understand best practice, options for coordination and lessons identified. In doing so, some of the challenges outlined above can be overcome to strengthen the science-policy interface for DRR.

## The Sendai Framework for DRR 2015-2030

On 18 March 2015, representatives from 187 UN Members States adopted the successor to the Hyogo Framework for Action and the first agreement of the post-2015 development agenda^8^. The Sendai Framework lays out an ambitious mandate for the next 15 years, with science firmly at the core of the priorities for action and in the principle of 'risk-informed decision-making' (paragraph 19 g). Learning from the lessons of the Hyogo Framework for Action 2005-2015, the Sendai Framework prioritises understanding disaster risk and strengthening governance at global and regional levels and recognises that this must involve application of risk information in all its dimensions.

Responsibility for enhancing scientific and technical work on disaster risk reduction is explicitly allocated to an enhanced UNISDR Science and Technical Advisory Group (STAG) (Paragraphs 19 g and 48 c). Table 1 presents the main points pertaining to international science and technology cooperation outlined in the Sendai Framework, particularly as it relates to the role of an enhanced STAG.

**Table 1. What the Sendai Framework says about international science and technology cooperation:** 

Paragraph	Statement text
**Priority 1**	**Understanding disaster risk**
25 (g)	Enhance the scientific and technical work on disaster risk reduction and its mobilization through the coordination of existing networks and scientific research institutions at all levels and all regions with the support of the UNISDR Scientific and Technical Advisory Group in order to: strengthen the evidence-base in support of the implementation of this framework; promote scientific research of disaster risk patterns, causes and effects; disseminate risk information with the best use of geospatial information technology; provide guidance on methodologies and standards for risk assessments, disaster risk modelling and the use of data; identify research and technology gaps and set recommendations for research priority areas in disaster risk reduction; promote and support the availability and application of science and technology to decision-making; contribute to the update of the 2009 UNISDR Terminology on Disaster Risk Reduction; use post-disaster reviews as opportunities to enhance learning and public policy; and, disseminate studies
**Priority 2**	**Strengthening governance and institutions to manage disaster risk**
27 (e)	Develop and strengthen, as appropriate, mechanisms to follow-up, periodically assess and publicly report on progress on national and local plans [...]
28 (a)	[...] create common information systems, and exchange good practices and programmes for cooperation and capacity development [...]
28 (b)	Foster collaboration across global and regional mechanisms and institutions for the implementation and coherence of instruments and tools relevant to disaster risk reduction, such as for climate change, biodiversity, sustainable development, poverty eradication, environment, agriculture, health, food and nutrition and others, as appropriate.
28 (c)	[...] to forge partnerships, periodically assess progress on implementation and share practice and knowledge on disaster risk-informed policies, programmes and investments, including on development and climate issues, as appropriate, as well as promote the integration of disaster risk management in other relevant sectors.
** Priority 3**	**Investing in disaster risk reduction for resilience **
31 (a)	Promote coherence across systems, sectors and organizations related to sustainable development and to disaster risk reduction in their policies, plans, programmes and processes.
31 (c)	Promote cooperation between academic, scientific and research entities and networks and the private sector to develop new products and services to help reduce disaster risk, in particular those that would assist developing countries and their specific challenges.
**Priority 4**	**Enhancing disaster preparedness for effective response and to "Build Back Better" in recovery, rehabilitation and reconstruction**
34 (b)	Promote the further development and dissemination of instruments, such as standards, codes, operational guides and other guidance instruments to support coordinated action in disaster preparedness and response and facilitate information sharing on lessons learned and best practices for policy practice and post-disaster reconstruction programmes
**Section VI**	**International cooperation and global partnership**
48 (c)	The United Nations Office for Disaster Risk Reduction (UNISDR), in particular, to support the implementation, follow-up and review of this framework through: preparing periodic reviews on progress, in particular for the Global Platform and, as appropriate, in a timely manner with the follow-up process at the United Nations, supporting the development of coherent global and regional follow-up and indicators and in coordination, as appropriate, with other relevant mechanisms for sustainable development and climate change and updating the existing Hyogo Framework for Action Monitor accordingly; participating actively in the work of the Inter-Agency and Expert Group on Sustainable Development Indicators: generating evidence-based and practical guidance for implementation in close collaboration with States, and through mobilization of experts; reinforcing a culture of prevention in relevant stakeholders, through supporting development of standards by experts and technical organizations, advocacy initiatives, and dissemination of disaster risk information, policies and practices, as well as providing education and training on disaster risk reduction through affiliated organizations; supporting countries, including through the national platforms or their equivalent, in their development of national plans and monitor trends and patterns in disaster risk, loss and impacts; convening the Global Platform for Disaster Risk Reduction and supporting the organization of regional platforms for disaster risk reduction in cooperation with regional organizations; leading the revision of the United Nations Plan of Action on Disaster Risk Reduction for Resilience; facilitating the enhancement of, and continuing services, the Scientific and Technical Advisory Group of the International Disaster Risk Conference in mobilizing science and technical work on disaster risk reduction; leading, in close coordination with States, the update of 2009 Terminology on Disaster Risk Reduction in line with the agreed terminology by States; and maintaining the stakeholders' common registry
50	The Conference recommends to the General Assembly the establishment at its sixty-ninth session of an open-ended intergovernmental working group, comprised of experts nominated by Member States, and supported by the United Nations Office for Disaster Risk Reduction (UNISDR), with involvement of relevant stakeholders development of a set of possible indicators to measure global progress in the implementation of this framework in conjunction with the work of the inter-agency expert group on sustainable development indicators. The conference also recommends that the Working Group considers the recommendations of the Scientific and Technical Advisory Group on the update of the 2009 UNISDR Terminology on Disaster Risk Reduction by December 2016, and that the outcome of its work be submitted to the General Assembly for its consideration and adoption.

## Methodology

In June 2014, an assessment framework was developed to learn from existing international science and technology partnerships in terms of their inception, governance, structure, operations, technical processes, and reporting (see Table 2). The domains included in this framework were guided by the DRR policy statements available at the time as part of the WCDRR preparatory work^12^^,^ 13, and by the typical areas of operation of existing international science partnerships to allow comparison.

The primary criteria for selection of partnerships for review were that the partnership operates at the international level, that it aims to bridge the science-policy interface to some extent and that the focus is on supporting disaster-related international issues, particularly including those to be discussed in 2015. The overarching objective has been to identify a broad range of models with differing roles, structures and procedures to learn from, rather than assessing an exhaustive list of the many science partnerships already in operation. Thus secondary criteria for inclusion were diversity of governance arrangements, of modes of operation and of processes, as well as differing reasons for establishment. First, the intergovernmental science partnerships supporting disaster-related international agreements were selected (e.g. IPCC, IPBES). Second, non-governmental international science partnerships were included (e.g. UNSDSN, RBM, SAB). Then a range of additional, diverse partnership models were included upon consultation with stakeholders. The full list of international science and technology partnerships reviewed is as follows:

- Intergovernmental Panel on Climate Change (IPCC)
- Intergovernmental Panel on Biodiversity and Ecosystem Services (IPBES)
- United Nations Sustainable Development Solutions Network (UNSDSN)
- Roll Back Malaria (RBM)
- Science Advisory Board of the Secretary General of the United Nations (SAB)
- Future Earth
- Global Framework for Climate Services (GFCS)
- International Agency for Research on Cancer (IARC)
- Scientific Knowledge for Environmental Protection (SKEP)
- United Nations Office for the Coordination of Humanitarian Affairs (UNOCHA) Cluster Coordination and United Nations Disaster Assessment and Coordination (UNDAC)
- International Oceanographic Commission (IOC)
- Climate Change and Clean Air Coalition (CCAC)
- International Decade for Natural Disaster Reduction (IDNDR)

A desk-based review of each partnership’s website, online documentation and independent evaluations (where available) was undertaken. Semi-structured interviews were conducted with 9 key informants, with close involvement with one or more partnership. Initial key informants were selected according to their roles in relation to international science partnerships, then a snowballing technique was employed in order to develop a more comprehensive list of key informants.

Interviews covered the broad themes identified in the analytical framework and were semi-structured, allowing for the interviewer to casually guide the general theme of the interview, with answers from interviewees being descriptive. Each interview lasted approximately an hour with points of view and key insights collated and transcribed.

The qualitative data collected from the desk-based review and interviews with key informants were then used to identify, classify and categorise common themes and sub-themes using thematic analysis^14^. The detailed review of partnerships can be found in Appendix 1.

**Table 2. List of questions for review:** 

QUESTIONS
**INCEPTION**
*What triggered the need for the partnership (e.g. a directive/particular problem)?*
*When was it established?*
*Who drove its establishment (e.g. a country/group of scientists)?*
*What are its objectives?*
*What have been the key challenges and barriers?*
**GOVERNANCE AND MANAGEMENT**
*Is the partnership intergovernmental?*
*Is the partnership independent?*
*Who comprises the members?*
*What is the structure?*
*What are the decision-making processes?*
*What are the election processes?*
*How is the partnership funded?*
**OPERATIONS**
*To what extent does the partnership collaborate with other initiatives?*
*To whom is the partnership accountable and how?*
*How does the partnership try to ensure transparency?*
*What are the key weaknesses in the structure/procedures? What has not worked so well?*
*What are the key strengths in the structure/procedures? What works well?*
**TECHNICAL PROCESSES**
*Who participates in providing technical information? Which stakeholders are included?*
*Does the partnership carry out new research?*
*What scales are considered?*
*Which types of knowledge are assessed?*
*Is there a monitoring and evaluation function?*
*How are reports/assessments reviewed?*
**REPORTING**
*What are the methods of reporting/dissemination?*
*Is there an advocacy/policy function?*
*Is there a capacity-building function?*
*Are there additional communications strategies in place?*
**EVALUATION**
*Has the partnership been formally evaluated?*
*Recommendations of evaluation?*
*If so, what changes have taken place as a result of the evaluation?*

## Review findings

The partnerships reviewed here cover a breadth of mandates and disciplines at the interface of science and technology with policy. These operate at the international level and aim to promote evidence-based policy to some extent.

The reasons for establishment of these partnerships vary. In some cases, the need to address a particular problem was driven by an increasing recognition of the problem by the political community, for example the IPCC was established as the concern over climate change became an increasingly political issue and the need for evidence to build consensus recognised^15 ^. Some were established to combine existing international processes, for example IPBES was conceived out of a coming together of the Millennium Ecosystem Assessment and International Mechanism of Scientific Expertise on Biodiversity (IMoSEB)^16^. Others have a convening purpose, e.g. Future Earth was established to fulfil the need for coordinated and solution-oriented scientific and societal response to global environmental change^17^, or engage very specific stakeholders, e.g. SAB of the Secretary General of the UN provides advice solely to the Secretary General and heads of UN organisations^18^, and UNSDSN aims to engage universities and academia in contributing to implementation challenges^19^.

It is worth noting that several of the partnerships reviewed here are new and currently in the early stages of their implementation (e.g. IPBES, UNSDSN and Future Earth) relative to more established partnerships like the IPCC, IARC, etc. In some cases, it is evident that these new partnerships are taking account of lessons identified in the implementation and evaluation of the earlier models, focussing on communications, actively collaborating with other partnerships and including a range of stakeholders in governance and the co-production of science^20^.

This section summarises the key themes emerging out of the analysis of material and interviews with key informants as important to the role, function and principles of international science partnerships.

***The science-policy interface***

Some partnerships have a research mandate, for example Future Earth aims for the co-production of knowledge^3^ and IARC carries out original research^21^, whereas others catalyse efforts to generate new knowledge, including a focus on solutions, working primarily through academic institutions^19^. IPBES does not undertake research but actively engages with Future Earth and other scientific organisations to catalyse new knowledge needed for policymakers at appropriate scales^22^. Several of the partnerships examined here have specific advocacy objectives. For example, RBM aims to implement coordinated action and forge consensus among partners^23^. As such, RBM has an Advocacy Working Group which aligns partner advocacy initiatives^24^. Similar to other partnerships, RBM does not carry out research, but supports research undertaken by partner organisations.

In contrast, IPCC is ‘policy-relevant and yet policy-neutral, never policy prescriptive’^25^. Governments play a role in agreeing the scope of scientific assessments, nominating authors and electing scientific leaders^26^. This level of political influence has led to challenges. For example, there has been criticism that the IPCC assessment process has become too closely associated with negotiations, whereby plenaries to agree scientific content have become increasingly politicised, a trend exacerbated by the funding procedures^27^. However, the IPCC is characterised by its global review process by scientists as well as governments, which is now publicly available on its website. This means that politicians cannot ignore the IPCC's findings. Two IPCC authors who were interviewed for this review felt that there is a role for policy recommendations from the Panel, as long as these are transparent, and suggest inclusion of a policy element to the partnership. Further challenges that have been cited for the IPCC with respect to the science-policy interface are: the sheer scale of work in terms of different interests involved and the nature of climate change as an issue (i.e. the uncertainty, multidisciplinarity and multiple levels of activity involved – local, national and international); the changing geopolitical context with growing influence of developing countries and the energy sector; rapid advances in climate science which has led to a greater number of authors on the IPCC reports and longer assessments; and, involvement of developing country experts and sources of knowledge^27^^,^ 28.

IPBES, which aims to strengthen the science-policy interface on biodiversity and ecosystem services, has followed the policy-relevant but not policy-prescriptive principle^22^, but it also actively supports policy formulation and implementation by identifying tools and methodologies to enable decision-making^29^. Further, the Multidisciplinary Expert Panel of IPBES is selected from 80% government-nominated experts and 20% stakeholder-nominated experts^30^. While the IPCC does not provide policy recommendations, IPBES produces deliverables in order to influence policy through advice with multiple options.

Other partnerships included in this review have clear science advisory roles. For example, the purpose for SKEP is to act as an international research platform to allow environment ministries, agencies and research councils within Europe to generate the evidence needed to underpin environmental regulations^31^. As such, SKEP produces rapid and concise responses to evidence queries, briefings for decision-makers and short reports to specifically address evidence gaps^31^. The SAB of the Secretary General of the UN was established to strengthen the interface between science and policy, forming part of the UN’s global strategy to mobilise the sciences to achieve the Millennium Development Goals and ensure integration of science into the post-2015 sustainable development agenda and to overcome the challenge that the science-policy relationship can be difficult and dysfunctional^18^. Contrary to other advisory bodies, the SAB is a body of independent scientists, which is deemed to be a key component of its added value. The need for the SAB was driven by recognition of the contentious nature of prominent scientific issues in recent years and a need to raise the visibility of science at the policy interface^18^.

The Cluster Coordination system and UNDAC perform an information management function ensuring that relevant information is available to partners during humanitarian crises and disasters^32^.

***Inclusivity, engagement and communications***

The partnerships reviewed here differ greatly in their approaches to membership, stakeholder engagement and the types of knowledge included in producing or assessing scientific information. Several are UN intergovernmental organisations with membership comprised only of UN member countries, e.g. IPCC and IPBES. While an intergovernmental approach helps to generate buy-in and funding for a partnership, this can also water down the policy implications of evidence generated^27^.

In an Inter Academy Council (now Partnership) evaluation of the IPCC undertaken in 2010, it was also recommended that a new Executive Committee be formed of members from private sector, academia, NGOs as well as IPCC leaders^26^. However, in implementation, this Executive Committee only includes IPCC leaders^25^. IPCC authors interviewed in this research agreed that it would be beneficial for business and NGO representatives to be included in the IPCC governance structure, if only to reduce the influence of governments on the structure and content of assessments^27^^,^ 28.

While membership is limited to UN member governments, IPBES encourages input from relevant stakeholders including governments, Multinational Environmental Agreements, NGOs, indigenous peoples, local communities, private sector, scientific community and UN agencies^33^. IPBES responds to requests and suggestions from this range of stakeholders, which are considered by Plenary to act upon^33^. Also in terms of provision of technical information, some partnerships are more inclusive than others, for example IPCC limits its assessments to consideration of science published in peer-reviewed journals. The IPCC has been criticised for its focus on knowledge held and generated in the global North. While efforts have been made to include more knowledge from the global South in recent assessments, the North-South divide is still perceived to exist^27^^,^ 34. While IPBES does incorporate different types of knowledge, the outputs produced by the Platform are peer-reviewed in an effort to retain scientific credibility^33^.

In contrast, UNSDSN is not an intergovernmental organisation, rather its membership comprises universities, research institutes, civil society organisations and other knowledge centres^35^. The intention is that these members act as partners in problem-solving and social entrepreneurs in SDSN’s Solution Initiatives^35^. Further, any individual can join UNSDSN via their website. Future Earth is essentially a global research programme and has representatives from a range of stakeholder communities including academia, funders, governments, international organisations and science assessments, development groups, business and industry, civil society and the media^35^. RBM includes 500 partners including governments of countries affected by malaria, development organisations, OECD donor countries, private sector, foundations, NGOs and CBOs, researchers and academics, all organised into 8 constituencies^36^.

Future Earth also has a dedicated Engagement Committee operating at a strategic level to ensure the body is a genuine platform for international engagement. The aim of this committee is to provide leadership and strategic guidance on involving stakeholders throughout the entire research process from co-design to dissemination, to help ensure Future Earth produces the knowledge that society needs^37^.

Communications is increasingly being recognised as an important component of science partnerships. Earlier partnerships do not tend to consider communications explicitly. One of the reported failings of the IDNDR was scientists underestimating the scale of the challenge in communicating science and technology to policymakers^38^. The IAC evaluation of the IPCC’s processes and procedures recommended a Communications Strategy be established to emphasise transparency, manage media relations and ensure rapid responses to queries^26^. This Strategy was adopted in 2012 with the goals to communicate assessment findings and methodologies, and to explain the way the IPCC works to promote understanding, transparency and credibility, particularly given the complex and politicised nature of the subject^39^. The IOC-UNESCO has grown closer to civil society in order to communicate effectively on ocean-related issues^40^.

Explicitly learning from the experiences of the IPCC, IPBES is currently developing a set of communication, outreach and engagement strategies, products and processes^6^^,^ 41. Science communication is acknowledged in the principles, and will be addressed through the implementation of the communication and stakeholder engagement strategy currently being developed for consideration by the Plenary^30^. UNSDSN does allow media and general enquiries to be submitted via their website but generally communication takes place mainly through personal contact and the SDSN website^35^. UNSDSN’s outputs aim to disseminate information to a wide audience via a range of outputs including reports, thematic group reports and issue briefs, which are published throughout the year^35^. These outputs are also open to public consultation.

RBM has a dedicated Communications Community of Practice Working Group within its structure, which aims to empower partners at the country level to develop, implement and evaluate effective communications activities^42^. An online media centre provides guidance and information.

***Governance structure***

The structure and governance of international bodies clearly poses a significant challenge. In several of the partnerships reviewed here, key informants have cited the difficulty of balancing the central Secretariat with peripheral organs as a weakness. For example, UNSDSN’s Solutions Initiatives are run as individual projects in a decentralised way while SDSN provides support through its Thematic Groups and network^35^. For GFCS, the heavy bureaucracy and regulations have been barriers to uptake by countries and the four sectors of the Framework do not coordinate well except at the country level^43^. Further, GFCS is not an entity or operational system so there are limited entry points at the national level. In an evaluation of RBM, the Board was considered too large and not representative of constituencies, contributing to a lack of country ownership^36^.

The IPCC has been criticised for being too bureaucratic and top-down in its processes^27^. This is partly attributed to the significant growth in climate science in the time since inception and the rapidly changing the scope and scale of work required in undertaking assessments. Greater flexibility and shorter reporting cycles have been recommended to overcome these weaknesses and promote more continuous dialogue between policy makers and the scientific community^27^. On the other hand, the UNSDSN has been challenged to find a balance between flexibility in structure and function and articulating a clear and common vision^35^.

The IAC evaluation of the IPCC recommended establishment of an Executive Committee to act on behalf of the Panel to strengthen and facilitate timely and effective implementation of work, to strengthen coordination between working groups and to address urgent issues that require prompt attention^26^. The design of IPBES procedures has been informed by the lessons identified in the IPCC evaluation^41^.

RBM has been evaluated several times, including in 2002 and 2013. In the former evaluation, tighter coordination was recommended to focus energies and improve accountability^44^. The Secretariat was found to need strengthening and more clearly defined technical roles for the WHO, which hosts RBM, and the Secretariat^44^. In the latter evaluation, the ‘structurally difficult’ hosting arrangement with WHO was highlighted with recommendation to revise^36^. Indeed, hosting arrangements are an important consideration. Most of the partnerships reviewed here are hosted by UN bodies, whether they are UN bodies or not e.g. RBM is hosted by WHO. An exception is UNSDSN which is run by the UN but is hosted by Columbia University and has no formal agreements with national governments^35^.

Under the Cluster Coordination system, responsibilities are necessarily flexible. Different UN bodies and/or NGOs take the lead depending on the cluster to be mobilised e.g. WHO leads the Health Cluster, FAO and WFP lead the Food Security Cluster, UNICEF and Save the Children lead the Education Cluster, and so on^32^. There is flexibility in which bodies lead action in any given crisis. For example, during mobilisation of the Health Cluster in Myanmar, there was no WHO representative so NGOs took the lead. In another instance, it may be WHO taking the lead. Where governments are strong, they tend to take the lead^32^. There have been difficulties in engaging partners to work together. For example, some country governments have not been engaged and some NGOs have not wanted to work closely with governments or UN agencies^32^. Local NGOs have not had as much of a voice where clusters mobilise and the clusters do tend to be more western-led and therefore western in their approaches^32^.

***Collaboration between partnerships***

As the number of international science partnerships grow and the importance of the science-policy interface comes to the fore, the need for collaboration across partnerships to avoid duplication and build on existing work is recognised. For example, Future Earth aims to develop a stronger and broader community by building on existing programmes including Diversitas, International Geosphere-Biosphere Programme (IGBP), International Human Dimensions Programme (IHDP), World Climate Research Programme (WRCP) and Earth System Science Partnership (ESSP)^3^. Future Earth and UNSDSN share board members.

In its founding principles, IPBES aims to collaborate with existing initiatives including the IPCC, Multilateral Environmental Agreements (MEAs), UN bodies and knowledge holders to fill gaps and avoid duplication^22^. In additional to participating in sessions of the Plenary, the Chairs of scientific subsidiary bodies of MEAs and the Chair of the IPCC are observers to the meetings of the IPBES Multidisciplinary Expert Committee^22^. It has also been requested that a collaborative partnership arrangement be formed with UNEP, UNESCO, FAO and UNDP^30^. Similarly, the SAB of the Secretary General includes in its board of experts the Chairs of IPBES and the IPCC and the Co-Chair of SDSN^45^.

For GFCS, the WHO, World Bank, UNDP, IFRC, UNISDR and FAO are all partners in principle and directly involved in the planning and implementation of GFCS-related activities in alignment with their mandates^46^. Other partners include the international NGOs and research institutes. The UNDAC participates with a range of UN agencies and economic bodies e.g. World Bank, ECOWAS and ASEAN^47^.

***Funding***

The conventional mode of funding for UN intergovernmental partnerships is voluntary contributions to dedicated trust funds. For instance, the IPCC receives regular contributions from WMO and UNEP as well as voluntary contributions from member states, a trust fund and direct state funding of working groups, technical support units *et cetera*.^48^. IPBES also has a trust fund open to voluntary contributions from all sources including governments, UN bodies, the Global Environment Facility, other intergovernmental organisations, the private sector and foundations^49^. The GFCS has a trust fund and receives in-kind member state donations and the United Kingdom Met Office and bilateral donors also contribute support^50^.

Others have more innovative funding partnerships. For example, UNSDSN received funding from individuals or foundations, the private sector, country government bodies and bilateral donors^51^. After initial funding from the European Commission Sixth Framework Programme, SKEP is now a subscriptions-based service to which only subscribed members can submit evidence queries^52^. IARC receives extra-budgetary resources for research through competitive grants from the Gates Foundation and European Commission for example, as well as participating state contributions^21^. The Cluster Coordination system is partially funded by NGOs if they are participating in response^32^.

Important lessons identified from the IPCC are that direct state funding of Working Groups and Technical Support Units has led at times to inequity and inevitable policy capture by individual governments^27^. A better approach suggested is to pool and share funding to promote shared ownership across the IPCC^27^. Nonetheless, direct funding of Working Groups can mean also that governments will commit funding to activities, and that those which do not can be conspicuous.

***Capacity building functions***

Approaches to capacity building differ widely across the reviewed partnerships. Some focus on human capacity building through training and education while others focus on building the institutional capacity of partners and countries. In terms of education and technical training, UNSDSN aims to accelerate joint learning with SDSN Assembly launched in 2014 to facilitate two-way flows of information between the Secretariat and members^53^. There is a dedicated SDSN Academic Committee to support the design and dissemination of educational materials^53^. Education and training is also a core component of IARC’s mission. The Agency provides fellowships and programmes of courses as well as making training an integral component of its research projects^54^.

One of the stated objectives of IPBES is to strengthen the capacity and knowledge foundations of the science-policy interface^55^. The Platform prioritises key capacity-building at appropriate levels within the global system then provides financial and other support for high-priority needs decided by the Plenary^55^. Similarly, Future Earth aims for increased capacity building in science, technology and innovation, especially in developing countries, and engagement with a new generation of scientists^17^.

The IPCC Task Group on Data and Scenario Support for Impact and Climate Assessment (TGICA) contributes to capacity building in the use of data and scenarios in developing and transition-economy regions and countries^56^. It does so by proposing a framework for training and overcoming capacity limitations that could be implemented by a third-party agency^56^.

The central aim of GFCS is to reinforce the capacity of national and regional institutions empowering them to deliver more accurate weather and climate services^57^. The intention is for National Meteorological and Hydrological Services to form national and international partnerships to enable the effective implementation of these activities. These should engage in national processes to mainstream these activities into national processes such as National Adaptation Plans, the UN Development Assistance Framework and so on^57^.

In a 2002 evaluation of RBM, it was recommended that a set of focus countries with high degrees of commitment be selected to make rapid progress and country champions be appointed for leadership in these countries^44^.

UNDAC aims to build common understandings and methods for coordination, information management and assessment among its members and partners^47^. Similarly, the Cluster Coordination system strives for a needs-based, rather than capacity-driven response to humanitarian crises, aiming to ensure coherent and complementary approaches among partners^58^.

***Monitoring and evaluation***

Based on the findings of the review, there are two kinds of monitoring and evaluation (M&E) of relevance to international partnerships. One is the routine M&E or auditing of the procedures and evidence produced at different levels (i.e. membership level or partnership level). The other is the undertaking of evaluative research and the capacity building required for this. This would include integrative studies on new approaches (e.g. humanitarian responses, disease treatments, early warning systems, *et cetera*.). A current example would be making Ebola a formal research priority. However, it is important to note that many of the partnerships reviewed here do not have M&E functions at all.

IPBES aims to review the effectiveness of guidance, procedures, methods and approaches to inform the future development of the Platform. The Multidisciplinary Expert Panel has been tasked with developing procedures for the review and effectiveness of administrative and scientific functions of the Platform^16^^,^ 29.

OCHA conducts internally- or externally-mandated evaluations to promote transparency, accountability and learning in the Cluster Coordination system^58^. All these evaluations are conducted by external experts and are carried out through systematic and objective judgements about the relevance, efficiency, effectiveness and impact of humanitarian interventions^58^.

RBM has an M&E Working Group, which facilitates the alignment of partners on strategies and ‘best practice’ for developing effective M&E systems, but does not carry out monitoring itself^59^. Country roadmaps monitor progress towards roadmap targets and RBM host this information and publishes Progress and Impact reports to benchmark process against global targets^59^.

## Lessons identified

It is important to give careful consideration to the role of international science partnerships with respect to the science-policy interface. In particular, the balance between the generation and assessment of science and technology needs to be well-defined in the objectives and practice of partnerships. In DRR there exists an important need to coordinate the many sources of information and synthesise these in a policy-focussed manner. Doing so may avoid the challenges faced by the IPCC or IDNDR, for examples. This is particularly the case since evidence-based DRR faces similar issues to climate science including the complex nature of the science, the geopolitical implications and the range of knowledge required for effective DRR.

Two coordinated and integrated processes are necessary for effective DRR. One is the generation of scientific evidence, which could involve strengthening the intergovernmental process around DRR. The other is for promoting the use of that science and technology to create evidence-based policy, through the influence and engagement of a network of academic and research institutions, UN bodies, private sector, NGOs and communities.

The Sendai Framework aims to knit the science-policy interface closer through 'generating evidence-based and practical guidance or implementation in close collaboration with States, and through mobilization of experts' (Paragraph 48 c). The stated role of an enhanced STAG to 'strengthen the evidence-base [... and] promote scientific research' encourages a role beyond assessment and advisory support, towards taking an active role to 'promote and support the availability and application of science and technology to decision-making' (Paragraph 25 g).

The findings of this review suggest that there should be allowance as much as possible for continuous engagement between scientists and policy makers in procedures and reporting, such that the needs of governments and other stakeholders can be met. The exact procedures by which an enhanced STAG will 'promote scientific research' and 'provide guidance' (Paragraph 25 g) are yet to be elucidated but will be important for the efficient functioning of international science cooperation for DRR.

This review has demonstrated that there is a range of new and established partnerships that have responsibilities and capacities for generating evidence of relevance to DRR. It is important that these sources of evidence are built upon, lines of communication strengthened and collaboration between across partnerships be achieved. An enhanced STAG should ensure procedures are in place to share in the generation of evidence across these partnerships and integrate sufficiently to avoid duplication and build on existing work, as is the shared aim of several of the partnerships reviewed here.

Further, the partnerships reviewed also have points of contact and procedures for engaging with a range of stakeholders including policymakers, the private sector, NGOs, civil society and others. Those established in recent years, e.g. Future Earth and UNSDSN, have tended to take more inclusive approaches to engagement, involving the private sector, local communities, UN bodies, academia and many other stakeholders. One of the cited achievements of the IDNDR was the bringing together of governments, NGOs and other international organisations to work with scientists. Now the challenge is to actively engage with a wide range of actors with a stake in DRR to elicit knowledge of different types and to effectively communicate evidence. Improved governance structure for an enhanced STAG must allow for the necessary levels of participation with new kinds of stakeholders, including the private sector and local communities.

The Sendai Framework performs relatively well on promoting inclusivity, engagement and communications. The Framework outlines the important role of an enhanced STAG in supporting the 'coordination of existing networks and scientific institutions at all levels and all regions' (Paragraph 25 g). The Framework text lists a comprehensive set of stakeholders (Section V), recognising the importance of partnership across member states, UN organisations, academic and scientific institutions, and of incorporating different types of knowledge, as held by indigenous and older people for example.

In terms of governance structure and processes, there is potential to consider flexible structures including voluntary working groups around key themes or issues of best practice. This approach need not provide a barrier to government participation but rather create opportunities to enable champions to engage in specific initiatives and get these started relatively quickly. This would help to establish best practices while providing for others to become involved at a later stage once the benefits are clear. A relevant example from this review is SDSN’s Solutions Initiatives, which have been taken up by additional governments once the evidence has been demonstrated.

Collaboration between partnerships must be also central to the functions of an enhanced STAG, specifically through the 'development of coherent global and regional follow-up and indicators and in coordination, as appropriate, with other relevant partnerships for sustainable development and climate change' (Paragraph 48 c). Active participation in the work of the Inter-Agency and Expert Group on Sustainable Development Indicators is specified (Paragraph 48 c). Overall there are few links to post-2015 agenda in the text and reference to the Sustainable Development Goals and UNFCCC agreements in the sections on implementation. Close collaboration between these and other partnerships will be necessary to achieve a successful outcome for the Sendai Framework, but will be difficult in practice. It will be the responsibility of an enhanced STAG to ensure that collaboration be transparent and consultative to maximise engagement of all stakeholders in these processes.

The review of other science partnerships demonstrates that private sector funding can be an important aspect for sustainable and inclusive action. In terms of the funding arrangements for UNISDR, an enhanced role for the United Nations Trust Fund for Disaster Reduction to assist with implementation is mentioned in the text (Paragraph 48 g). Strengthening the Trust Fund could pool funding for UNISDR, including towards assisting an enhanced STAG with its expanded mandate. While country contributions and UN support will be an important element of the Sendai Framework, new modalities for leveraging private sources of funding should also be used to divert finance streams to the enhanced STAG, particularly given the close links between parts of the private sector and DRR (e.g. the insurance and construction industries). The role of business, professional associations and private sector financial institutions to implement disaster risk-informed investments is there (Paragraph 36 c). Such investments could include for work to support the functions of the enhanced STAG. Again, it is critical that funding arrangements be as transparent and inclusive of as possible such that all stakeholders, particularly developing countries, have a stake in the success of UNISDR and an enhanced STAG.

Based on the findings of this review, there are several approaches that can be taken to build capacity for evidence-based policy at the international level. These include providing training for a new generation of leaders or scientists (e.g. SDSN, IARC), providing technical training on specific issues in response to identified gaps and needs (e.g. IPCC TGICA), or coordinating the capacity of national and regional bodies or institutions for (e.g. GFCS, IOC). The ways in which capacity can be built should be decided in consultation with stakeholders to ensure capacity building objectives match with perceived needs. It appears to be important for achieving capacity-building objectives to clearly identify the link between need and action and get the buy-in of those involved. It is also important to recognise the flexibility required to respond to differing needs.

An enhanced STAG is also to play a role in the 'dissemination of disaster risk information, policies and practices, as well as providing education and training on disaster risk reduction through affiliated organizations' (Paragraph 48 c). This statement implies a capacity building function for an enhanced STAG. Additionally, there are priorities to 'strengthen technical and scientific capacity to capitalize on and consolidate existing knowledge' at national and local levels (Paragraph 24 j) and 'to create common information systems and exchange good practices and programmes for cooperation and capacity development' at global and regional levels (Paragraph 28 a). Overall, these provisions stress the importance of capacity building at all levels in reducing disaster risk and suggest an enhanced STAG must facilitate this.

Based on the findings of this review, M&E are relatively unusual function for international science partnerships. Where M&E has been explicitly addressed in the partnerships reviewed here, it has been focussed mostly on auditing the procedures and outputs of the partnerships themselves. RBM works to align the strategies and practice of partners to meet globally-established malaria eradication targets.

There is recognition that the Hyogo Framework did not meet set targets in all regions or countries and lack of scientific evidence and uptake of evidence is one of the reasons for this. The Sendai Framework promotes 'strengthening of [...] international voluntary partnerships for monitoring and assessment of disaster risks, including relevant data and information' (Paragraph 28 f). It also gives UNISDR the mandate to support countries to 'monitor trends and patterns in disaster risk, loss and impacts' (Paragraph 48 c), citing the importance of the Hyogo Framework for Action Monitor in achieving this. An enhanced STAG will play a key role in ensuring data is collected in a standardised way against the targets and indicators of the Framework across all member states.

## Conclusions

Science clearly plays an important role at the international level in informing policy on key issues such as climate change, biodiversity and ecosystem protection and sustainable development. The inception of new science and technology partnerships in the past few years indicates a regeneration of science advisory services at the international level, recognising that scientific inputs are only one component of the science-policy interface. Partnerships such as IPBES and UNSDSN recognise the need to build the capacity of different kinds of actors, including policy makers and scientists, and for two-way, continuous communication, mediated by boundary organisations within the evidence-based approach to policy making.

The need for an improved science-policy interface and evidence-based approach to DRR has been recognised by UN member states in the Sendai Framework for DRR. The Framework provides a comprehensive list of functions for incorporating science and technology in future DRR action. This is to be commended and is a demonstration of commitment from the international community for evidence-based decision-making in facing the challenges of the post-2015 era. What is missing from the agreement is clarity on what an enhanced STAG might look like in terms of governance processes and structure. These arrangements will have implications for how UNISDR can implement the aspirations of member states. It will be necessary to prioritise actions and execute these rapidly to achieve a successful outcome by 2030.

The intergovernmental working group (Paragraph 50) should play a key role in setting the groundwork for an enhanced STAG, ensuring specific governance structures and processes are in place by 2016. The precise functioning of the interface with the wide range of policy makers at Member State and international level should be agreed in partnership in as transparent and flexible a way as to meet user needs. The principles of inclusivity and engagement captured in the Framework text must be enforced, incorporating the full range of stakeholders into the provision of scientific and technical information and building capacity for those who currently lack the ability to do so. The innovative funding modalities employed by more recent international science partnerships should be considered and employed so as to ensure all stakeholders truly have a stake in effective future international science cooperation. Every step of this process must be as open, as inclusive and as participatory as possible in order to build trust in new and existing institutions at local, national, regional and global levels.

Lastly, 2015 offers a unique window of opportunity to improve international governance around climate change, disasters, development finance and sustainable development. Practical ways to achieve policy coherence in the post-2015 era include shared targets and indicators across frameworks, coordinated monitoring of progress, collaboration in sharing information and in common financing partnerships. A revitalised international partnership for evidence-based DRR can help to deliver this promise. A starting point will be through 'the development of a set of possible indicators to measure global progress in the implementation of this Framework in conjunction with the work of the inter-agency expert group on sustainable development indicators' (Paragraph 50). Beyond this, an enhanced STAG can play an important role to 'foster collaboration across global and regional mechanisms and institutions for the implementation and coherence of instruments and tools relevant to disaster risk reduction, such as for climate change, biodiversity, sustainable development, poverty eradication, environment, agriculture, health, food and nutrition and others, as appropriate' (Paragraph 28 b). The challenge for the international community is to facilitate such collaboration by formally recognising the links in the upcoming post-2015 agreements.

## Appendix 1

### Review of international science partnerships

The tables in the PDF present the findings of the review of the international science partnerships listed in the methodology. The information provided in these tables was provided through desk-based review of organisation websites, related publications and also by consultation with key informants. This represents the best available information within the bounds of this research, and in places reflects the judgements of the author and key informants who participated in this research. Download PDF
